# Current News

**Published:** 1897-09

**Authors:** 


### Current News.

### NEW JERSEY STATE DENTAL SOCIETY.

At the Twenty-seventh Annual Meeting of the New Jersey State Dental Society, held July 21, 22, and 23, 1897, at Atlantic City, N. J., the following officers were elected:

President, Dr. J. L. Crater, Orange; Vice-President, Dr. J. Allen Osmun, Newark; Secretary, Dr. Charles A. Meeker, Newark; Treasurer, Dr. H. A. Hull, New Brunswick.

*Executive Committee.*—Dr. H. S. Sutphen, Newark; Dr. Oscar Adelberg, Elizabeth; Dr. F. E. Riley, Newark; Dr. C. W. F. Holbrook, Newark.

*Membership Committee.*—Dr. William E. Truex, Freehold; Dr. F. L. Hindle, New Brunswick; Dr. F. G. Gregory, Newark; Dr. William L. Fish, Newark; Dr. William H. Pruden, Paterson.

